# Assumption-Free Estimation of Heritability from Genome-Wide Identity-by-Descent Sharing between Full Siblings

**DOI:** 10.1371/journal.pgen.0020041

**Published:** 2006-03-24

**Authors:** Peter M Visscher, Sarah E Medland, Manuel A. R Ferreira, Katherine I Morley, Gu Zhu, Belinda K Cornes, Grant W Montgomery, Nicholas G Martin

**Affiliations:** Genetic Epidemiology Group, Queensland Institute of Medical Research, Brisbane, Australia; North Carolina State University, United States of America

## Abstract

The study of continuously varying, quantitative traits is important in evolutionary biology, agriculture, and medicine. Variation in such traits is attributable to many, possibly interacting, genes whose expression may be sensitive to the environment, which makes their dissection into underlying causative factors difficult. An important population parameter for quantitative traits is heritability, the proportion of total variance that is due to genetic factors. Response to artificial and natural selection and the degree of resemblance between relatives are all a function of this parameter. Following the classic paper by R. A. Fisher in 1918, the estimation of additive and dominance genetic variance and heritability in populations is based upon the *expected* proportion of genes shared between different types of relatives, and explicit, often controversial and untestable models of genetic and non-genetic causes of family resemblance. With genome-wide coverage of genetic markers it is now possible to estimate such parameters solely within families using the *actual* degree of identity-by-descent sharing between relatives. Using genome scans on 4,401 quasi-independent sib pairs of which 3,375 pairs had phenotypes, we estimated the heritability of height from empirical genome-wide identity-by-descent sharing, which varied from 0.374 to 0.617 (mean 0.498, standard deviation 0.036). The variance in identity-by-descent sharing per chromosome and per genome was consistent with theory. The maximum likelihood estimate of the heritability for height was 0.80 with no evidence for non-genetic causes of sib resemblance, consistent with results from independent twin and family studies but using an entirely separate source of information. Our application shows that it is feasible to estimate genetic variance solely from within-family segregation and provides an independent validation of previously untestable assumptions. Given sufficient data, our new paradigm will allow the estimation of genetic variation for disease susceptibility and quantitative traits that is free from confounding with non-genetic factors and will allow partitioning of genetic variation into additive and non-additive components.

## Synopsis

Quantitative geneticists attempt to understand variation between individuals within a population for traits such as height in humans and the number of bristles in fruit flies. This has been traditionally done by partitioning the variation in underlying sources due to genetic and environmental factors, using the observed amount of variation between and within families. A problem with this approach is that one can never be sure that the estimates are correct, because nature and nurture can be confounded without one knowing it. The authors got around this problem by comparing the similarity between relatives as a function of the exact proportion of genes that they have in common, looking only within families. Using this approach, the authors estimated the amount of total variation for height in humans that is due to genetic factors from 3,375 sibling pairs. For each pair, the authors estimated the proportion of genes that they share from DNA markers. It was found that about 80% of the total variation can be explained by genetic factors, close to results that are obtained from classical studies. This study provides the first validation of an estimate of genetic variation by using a source of information that is free from nature–nurture assumptions.

## Introduction

The theoretical basis for the resemblance between relatives due to genetic factors was developed by R.A. Fisher in a now famous and classic paper that reconciled Mendelian and biometrical genetics [1]. Following that theoretical basis, quantitative genetic parameters are estimated from the resemblance between different types of relatives by equating the observed phenotypic covariance to the degree of genetic relationship, which is estimated from pedigree data. The degree of relationship is usually expressed as the coefficient of kinship [2] or the additive coefficient of relationship [2,3]. In a non-inbred population, the coefficient of relationship is the expected proportion of alleles identical-by-descent (IBD) between relatives and determines the additive genetic covariance between a pair of relatives. Maximum likelihood (ML) methods and software have been developed to estimate genetic (co)variances in simple [4] and large complex pedigrees [5–7], for univariate and multivariate models. What all these methods have in common is that they estimate genetic parameters from observed variation between and within families, assuming an underlying model for causative components of variance [3]. For example, in twin studies it is commonly assumed that the variance between families is due to common environmental and additive genetic effects, and that the variance within families reflects individual environmental effects (for monozygotic [MZ] pairs) or both individual environmental and additive genetic effects (for dizygotic [DZ] pairs).

In human populations, the interplay of genetic, environmental, and cultural factors that cause family resemblance is complex; and crucially, the ultimate separation of nature and nurture effects can generally not be tested empirically through controlled experiments. If the true (unknown) effects causing between-family variance deviate from the assumed model of family resemblance, then the resulting estimates of genetic parameters, and their estimated standard errors (SE), will be biased. This bias could be severe if strong assumptions are necessary to estimate genetic parameters. In the classical twin design, only three underlying parameters are estimated, and strong assumptions regarding the causes of familial resemblance are necessary. For example, the assumption that twin resemblance due to common environmental effects is the same for MZ and DZ pairs is often made. Although some of these assumptions can and have been tested empirically [8,9], the use of twin data to estimate heritability, in particular for traits such as cognitive function, has been controversial [10].

Until now, it has been impossible to exclude a possible confounding between genetic and non-genetic causes of family resemblance. We propose an alternative approach to estimate genetic variance that is based upon the observed proportion of the genome that is shared by relatives and does not make any assumptions about the variation between families.

The actual genome-wide relationship, defined as the proportion of the genome that two relatives share IBD, varies around its expectation because of Mendelian segregation [11–14], except for MZ twins and parent-offspring pairs. We use the term “actual” throughout, but other possibilities are “realized relationships” or “the proportion of the genome-shared IBD.” It is possible to estimate this relationship with the use of genetic markers. If these estimates are accurate, then it is, in principle, feasible to estimate genetic parameters within families, obviating the need for contentious assumptions about the sources of between-family variation.

In this study, we estimated heritability for height in humans without making any assumptions regarding the causes of resemblance between relatives. We present the relevant theory and estimate the heritability of height from collections of 3,375 full-sib pairs, using genome-wide estimates of actual additive genetic relationships. Bias and accuracy of our estimation approach was explored analytically and by computer simulation. Ours is the first example of an estimate of heritability in humans for which a possible confounding between nature and nurture can be excluded.

## Results

### Simulated Data

We first assessed bias and accuracy of the estimates of variance components from our method using simulation studies and analytical predictions (see Materials and Methods). Table 1 shows the empirical mean and SE of the ML estimate of the heritability from actual relationships between sibling pairs and statistical power, and their theoretical predictions, for a range of population parameters. As predicted by theory (see Materials and Methods), the SE of the estimates are large, unless the number of pairs is large (10,000), the heritability is large (>0.6), or there is no residual family effect. For 2,500 sib pairs, the SE of the heritability is approximately 0.2 when the true value is 0.8. For 10,000 sib pairs, the range of SE is from 0.08–0.19. The theoretical predictions are accurate, in particular for the special case when the proportion of variance due to non-genetic family effects *(f^2^)* is zero. When the proportion of variance due to non-genetic family effects is zero, the estimate of the heritability is biased downwards, in particular when the sample size is small (Table 1). This is because we constrain variance components to be non-negative in our ML estimation procedure. An analytical prediction of the bias is given in Materials and Methods. When the heritability is large (0.8), its estimate is biased downwards, even when the proportion of variance due to non-genetic family effects was larger than zero. Again this is the result of ML estimation, because the sum of the proportion of variance due to genetic and non-genetic factors cannot be larger than unity.

**Table 1:**
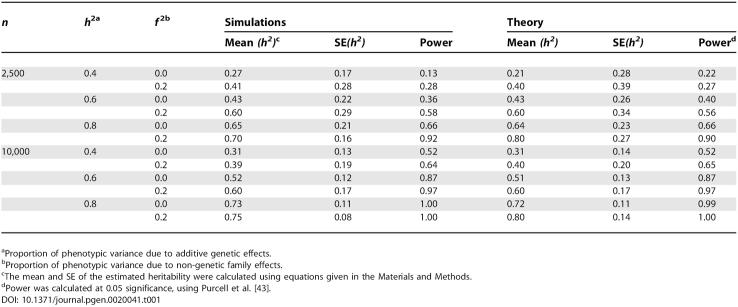
Predicted and Empirical Power and Standard Errors of Heritability Estimation from Genome-Wide IBD Sharing between Sib Pairs

### Data Application

There were a total of 4,401 quasi-independent sibling pairs with estimates of genome-wide IBD sharing statistics. The average proportion of the genome-shared IBD between the sib pairs (the coefficient of additive genetic variance) was 0.498 (SE 0.0005, standard deviation [SD] 0.036), with a range of 0.374–0.617. The distribution of the genome-wide additive coefficients is shown in Figure 1. The mean and range of the proportion of the genome for which a sibling pair shared two alleles IBD (the coefficient of dominance variance, also termed IBD2) was 0.248 (SE 0.0006, SD 0.040) and 0.116–0.401, respectively. Hence, both mean sharing statistics were slightly lower than the expected values of 0.50 and 0.25, respectively. When comparing the mean sharing statistics to their SE, there was evidence for a small but significant departure from expectation (*p* = 0.002 and 0.0002, for genome-wide additive and dominance coefficients, respectively, assuming a normal distribution of the test statistic). However, the SE is under-estimated because not all pair-wise sib comparisons are independent, so that the departure from expectation is less significant than it appears from the reported *p*-values. The SD of the mean (additive) IBD and mean IBD2 (dominance) sharing proportions were 0.036 and 0.040, respectively. One quality control measure of our IBD calculations is to test for independence of chromosome-specific additive and dominance relationships. For the combined dataset, 8/231 and 2/231 Spearman rank correlations of the mean IBD sharing between chromosomes were significant at the 0.05 and 0.01 level, respectively, when 12 and 2 were expected under the assumption of independent segregation. For IBD2 sharing the corresponding numbers were 9/231 and 1/231. The observed numbers are not significantly different from expectation under the null hypothesis of independent segregation of chromosomes (the SD of the number of significant correlations at the 0.05 and 0.01 level under the null hypothesis is 3.3 and 1.5, respectively).

**Figure 1:**
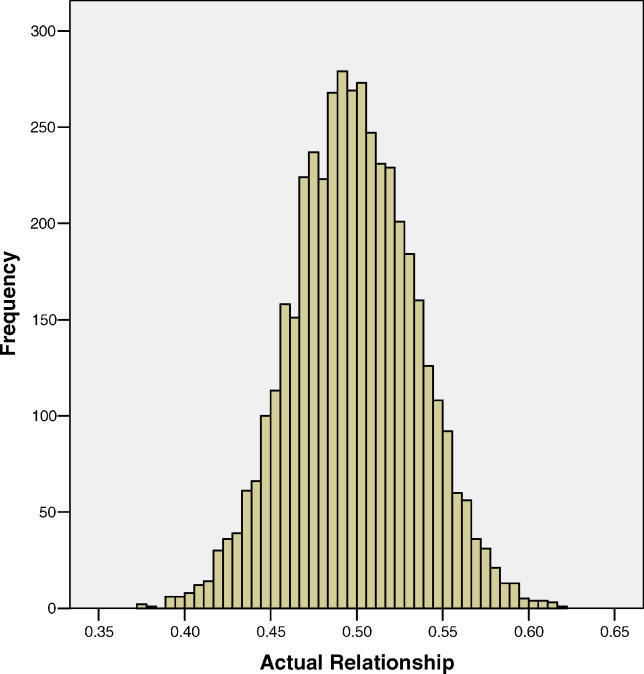
Empirical Distribution of Actual Additive Genetic Relationships of 4,401 Quasi-Independent Pairs of Full Sibs Histogram of the genome-wide additive genetic relationships of full-sib pairs estimated from genetic markers.

Figure 2 shows the empirical variance of genome-wide mean IBD and IBD2 sharing, relative to the expected value from theoretical considerations (see Materials and Methods). There is a remarkably good agreement between theory and data, with a correlation between the theoretical and empirical SDs across chromosomes of 0.98 for both mean IBD sharing and mean IBD2 sharing. The correlation between mean IBD and mean IBD2 sharing for 4,401 pairs was 0.91, close to the theoretical value of 0.89 (Figure 3). This large correlation implies a strong sampling correlation between the estimates of additive and dominance variance.

**Figure 2:**
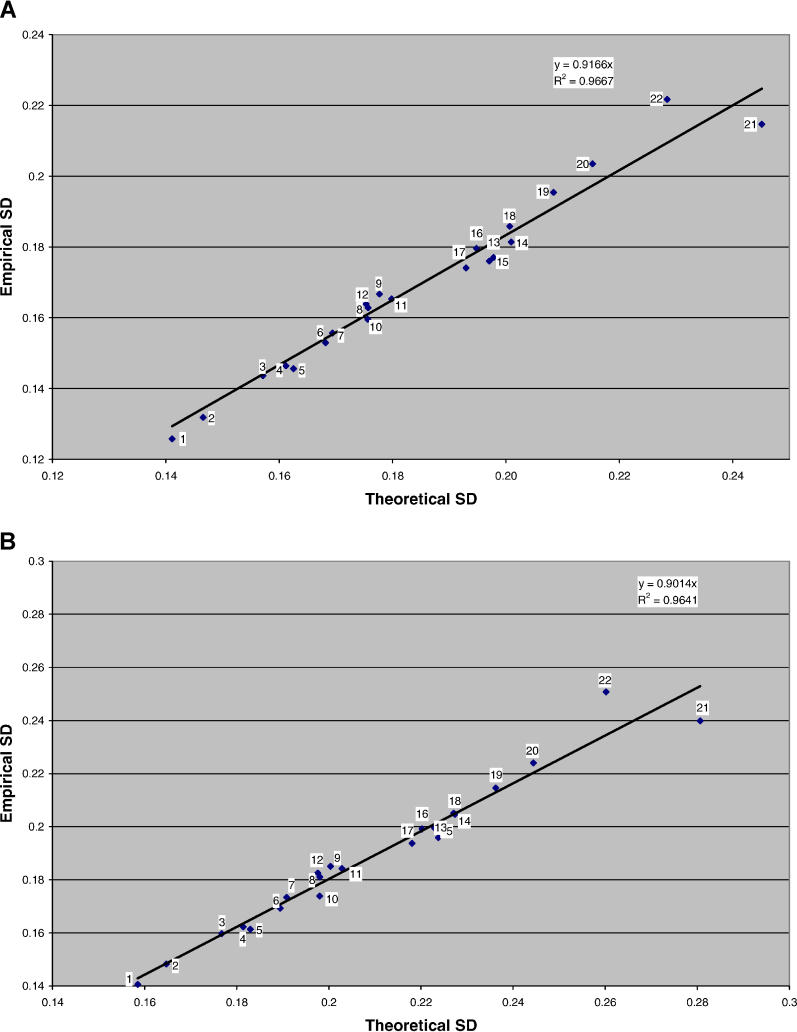
Comparison of the Empirical and Theoretical Standard Deviations in Genome-Wide Sharing in 4,401 Quasi-Independent Pairs of Full Sibs IBD sharing (A) and genome-wide IBD2 sharing (B) from marker data on 22 autosomes. The *x*- and *y*- axes are the theoretical and empirical SD, respectively. Numbers around the regression line indicate chromosomes.

**Figure 3:**
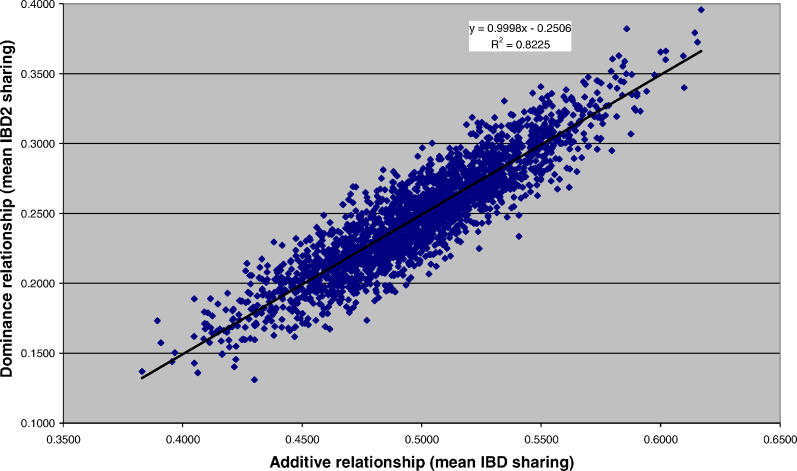
Correlation between Genome-Wide Mean IBD and Mean IBD2 Sharing The *x*- and *y*- axes are the genome-wide additive and dominance relationships, respectively. Each point represents the genome-wide additive and dominance relationship for a sibling pair, estimated from genetic markers (*n* = 4,401 pairs).

ML estimators of heritability are shown in Table 2 for the two datasets separately and for the combined dataset. For each dataset, two models were fitted: a full model (FAE), containing a non-genetic family effect (F), a genome-wide additive effect (A), a residual error effect (E); and a reduced model containing F and E effects only (FE). In all analyses, the estimate of the residual family component was zero, and the estimate of heritability 0.8. For the combined dataset (*n* = 3,375 pairs), the 95% confidence interval (CI) was from 0.46 to 0.85 (a SE of approximately 0.1) with strong statistical support (*p* = 0.0003) for a variance associated with genome-wide IBD. The SE of the estimated proportion of variance due to additive genetic variance *(h^2^)* is large relative to the estimate. However, because the sampling correlation of the estimates of the non-genetic and genetic variance is large and negative, the estimate of the total proportion of variance explained by genetic and non-genetic effects (i.e., the predicted MZ correlation) is more accurate. For the combined dataset, the ML estimate of this proportion is 0.80, with a 95% CI of 0.62–0.85. Hence, we have estimated the equivalent of an MZ correlation without having such pairs in our data.

**Table 2:**
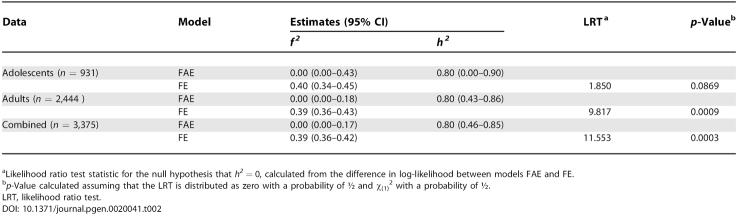
ML Estimates of Heritability of Height from Genome-Wide IBD Sharing between Sib Pairs

The estimates from the FE model reflect the sibling correlation of 0.40 and 0.39 for the adolescent and adult datasets. Estimates of the proportion of variance due to additive genetic effects from the AE model (not shown in Table 2) were very close to twice the estimates of the proportion of variance due to the family effect in the FE model.

When genome-wide dominance was fitted in addition to F and A, the log-likelihood did not increase significantly for the combined dataset (unpublished data). However, there is unlikely to be sufficient power to distinguish these components with our sample size, consistent with our observed correlation coefficient of 0.89 between the additive genetic and dominance coefficients (Figure 3).

## Discussion

We have shown that it is feasible to estimate genetic parameters solely from segregation within families, without making any assumptions regarding an underlying model for between-family effects. In fact, our only assumption in the analysis is that the additive genetic covariance between relatives is proportional to the actual proportion of the genome that is shared IBD. The resulting estimates of the heritability for height (0.80, 95% CI, 0.46–0.85) and residual family effects (0.00, 95% CI, 0.00–0.17) are very close to estimates from twin studies [15], where the information comes from the difference in correlation between MZ and DZ twin pairs. Essentially, we have estimated the same parameters from DZ and full-sib pairs only.

Previously, methods have been proposed to estimate kinship and genetic parameters from marker data when pedigree data are not available, for example, in natural populations [16–18]. Relationship estimation and reconstruction in these methods are based upon identity-by-state sharing of marker alleles. These methods have the same principle as our approach, i.e., first estimating kinship from marker data and subsequently estimating genetic variance from the association between phenotype similarity and estimated kinship. However, there are some important differences between the methods. Firstly, our method is based upon IBD sharing, i.e., we know the pedigree and estimated actual relationships from marker data conditional on the pedigree. The resulting estimates of actual kinship are unbiased and have lower error variance, provided that the pedigree is correct. Secondly, we estimate genetic variance free from possible confounding with environmental factors. In natural populations, even if the kinship were to be estimated without error, there can still be a confounding between genetic and environmental similarities and this could lead to bias.

We do not suggest that all estimation of genetic (co)variance from classical designs that utilize between-family comparisons should be abandoned. On the contrary, such designs, for example, those employing twin families, are in principle powerful enough to separate genetic and non-genetic causes of family resemblance if the statistical models are correct or at least a good approximation of the true underlying causes of variation. With sufficient data, our approach allows the testing of hitherto untestable underlying assumptions in other models and, for large samples, allows the estimation of non-additive genetic variation for disease susceptibility and quantitative traits. Therefore, the two methods should be seen as complementary.

There is a continuum in the estimation of genetic parameters from genome-wide IBD sharing to quantitative trait loci (QTL) mapping. In QTL mapping, variation in IBD sharing is maximal but many estimations/tests are performed. For sib pairs, the variance of IBD sharing at a single location is ^1^/_8_ [14,19–21], whereas it is only 0.039^2^ genome-wide. Hence, relative to the mean there is about 82 times more variation in IBD sharing between sib pairs at a particular locus than in the genome-wide average [22]. The disadvantage of QTL mapping is that a genome-wide search is performed at many correlated locations, whereas the estimation of genetic variance from genome-wide IBD sharing is a single estimate. An intermediate between the two is to estimate the proportion of additive genetic variance associated with a chromosome [23–25]. The variance in proportion of a chromosome-shared IBD is intermediate between the sharing proportion at a single location and genome-wide, as shown in Table 3. We note that the emphasis in this study is on the estimation of genetic parameters rather than its detection. Hence, in contrast to QTL mapping where hypothesis testing and *p*-values are important, we have concentrated on the sampling variance of the estimated parameters, because for most traits it is usually known that there is genetic variance, and the scientific question is what proportion of observed variation is genetic.

**Table 3:**
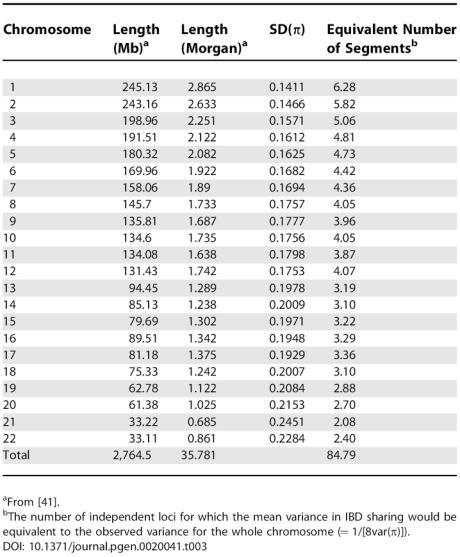
Theoretical Standard Deviation of IBD Sharing between Sibs per Chromosome

Although our estimates of the variation in mean IBD and IBD2 sharing per chromosome are very similar to the theoretical values (Figure 2) and consistent with recently reported genome-wide sharing statistics from a sample of 498 sib pairs [22], a few caveats are required. Firstly, the theoretical value may be too low for the true variance in IBD sharing on a chromosome because in reality there may be more crossovers than modeled [13,14]. Secondly, the empirical variance of IBD sharing is likely to be an underestimate because the marker information was not perfect. If we assume that our genome-wide average multipoint marker information content was approximately 80%, then we would expect to find a regression slope of the empirical on theoretical SD in IBD sharing of √ 0.80 = 0.89, close to the observed value of 0.92 (Figure 2A). Nevertheless, the correlations of 0.98 between empirical and theoretical values are extremely high.

We detected a small genome-wide deviation of the observed IBD sharing statistics from expectation. Genome-wide transmission distortion, which results in excess allele sharing between relatives, has been reported previously [26]. Our results were driven by a deficit of the probability of sharing two alleles IBD, hence we do not replicate the findings of [26] with our large sample of 4,401 pairs.

Our simulation studies confirmed that a large number of pairs is needed for accurate estimation, and showed that the estimates of heritability were biased downwards when there was no underlying source of non-genetic family resemblance. This bias is the result of ML estimation because of the usual constraints that estimated variance components have to be non-negative and that the sum of the partitioned variance ratios is bounded by zero and one. The observed bias is not particular to our method because it applies to any variance partitioning approach by ML, in particular when sampling variances are large [27,28].

We have estimated a single additive genetic variance from genome-wide segregation of marker loci within families, after adjusting phenotypes for the fixed effects of sex and age at measurement. However, genetic variances for males and females and younger and older siblings may be different, and the genetic correlation across these groups may be smaller than unity. Although we have ignored these potential sources of heterogeneity of genetic variance in this study because of sample size considerations, models that include, for example, sex-limitation effects are, in principle, straightforward to implement.

We have ignored the contribution of the sex chromosomes to genome-wide IBD. In humans, the X chromosome accounts for 4% of genes and 5% of physical length [29]. If all chromosomes account for genetic variation in proportion to the number of genes or physical length, then our estimate of heritability will be biased downwards by about 4% to 5%.

Although our sample size of 3,375 was sufficient to estimate the heritability of height with reasonable accuracy, for phenotypes with smaller heritability (and to distinguish additive from dominance variance), larger sample sizes are necessary. Such large datasets are in the process of being generated, either from large national studies or by combining samples across countries. For example, the GenomEUtwin study will accrue over 10,000 sib pairs for linkage studies [30]. Therefore, in the near future we will be able to estimate unbiased genetic parameters for traits that have been controversial in the past due to the assumptions regarding the (non-genetic) resemblance between relatives. If a large population resource of relatives with measured phenotypes were to be available, then a selective genotyping strategy in which only concordant and discordant pairs are genotyped may be efficient in estimating quantitative genetic parameters accurately, for the same reason that such a design can be powerful in gene mapping studies [31,32].

Our application was on a single quantitative trait and using a simple pedigree structure. However, the method is entirely general and can be applied to disease phenotypes, multiple traits, and large arbitrary pedigrees. All that is required is genome-wide estimates of IBD sharing between relatives, observations on relevant phenotypes, large samples, and software to estimate components of (co)variance.

There are limitations of the applied method, the main one being that large sample sizes are required with dense marker coverage of genotyped individuals. This may be unachievable for most single labs now, but future large population-based studies that have a family component, or pooling of sample resources across studies, will have the desired effect of increasing sample size. A second limitation is that sufficient markers need to be genotyped to obtain an accurate estimate of genome-wide sharing statistics. This is less of a problem because many samples that are suitable for our suggested analyses are genotyped for linkage studies, and marker density is likely to increase in the near future because of the availability of relatively cheap single nucleotide polymorphism genotyping. With the advent of high density single nucleotide polymorphism genotyping platforms, the error in estimation of genome-wide IBD sharing between relatives is likely to be small, and we have assumed, in the present study, that it is negligible. If the estimation of genome-wide IBD sharing is less than 100% accurate, then the variation in IBD sharing between pairs is less than the true variation, resulting in less powerful analysis but still unbiased estimates [33]. With less complete marker coverage, the estimate of the proportion of alleles shared IBD is unbiased 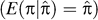 but has larger prediction error variance. For a single location in the genome, we derived the prediction error variance as: 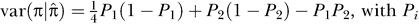 , with *P_i_* the probability of having *i* alleles IBD; note that this variance could be used as a weight in gene mapping studies. To a first order approximation, the sampling variance of the estimate of the heritability, relative to the situation of perfect marker information, is increased by the reciprocal of the average genome-wide information content [34]. A third limitation is that it is difficult to disentangle additive from non-additive effects. However, with sufficient data the large correlation between additive and dominance coefficients is not an issue, and one could even consider estimating additional non-additive effects, for example additive-by-additive or additive-by-dominance effects.

In conclusion, we have shown that it is feasible to estimate genetic variance entirely within families, by correlating phenotypes and genome-wide similarity. Our assumption-free method facilitates a complete separation of genetic and environmental causes of family resemblance and will allow the estimation and testing of non-additive sources of variation.

## Materials and Methods

### Variance of genome-wide IBD sharing.

The variance of the proportion of chromosome segments that are IBD between relatives has been derived by a number of authors for pairs of full sibs [13,14,23,33,35], complex pedigrees [12,36,37], for inbred individuals [38], and for experimental backcross populations [39,40]. In the case of full sibs we give a derivation for both the additive and dominance component of covariance, and their correlation, following the approach of Hill [39].

### Additive effects.

For a given sib pair, the genome-wide mean IBD sharing (π) is the sum of the proportion shared from the paternal (p) and maternal (m) contribution,


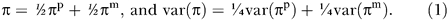


Hence, to calculate the variance it is sufficient to consider the contribution from a single parent only. For parent k, the sharing of alleles by progeny depends on the proportion of alleles shared due to the parent's paternal or maternal gamete. Let δ_i_ be an indicator variable for locus i, which is one if both sibs have inherited the paternal allele or both sibs have inherited the maternal allele, and zero otherwise. Then,


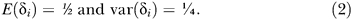


The covariance of the indicator variables at two loci (i and j) is:


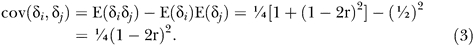


Assuming the Haldane mapping function, the covariance can be written as:


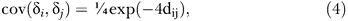


with d_ij_ the distance (in Morgan) between the loci. For *n* loci, the variance of chromosome-wide sharing between two sibs is:


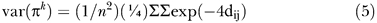


(following [39]). If *n* becomes very large this equation can be expressed as an integral [12,39], 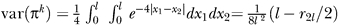 with *l* the length of the chromosome (in Morgan) and r_2*l*_ the recombination fraction for a segment of length 2*l.* Hence, the total variance in IBD sharing between two siblings for chromosome i of length *l* is:


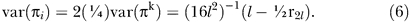


Finally, genome-wide π is, π_g_ = (1/L) Σ(*l*_i_ π*_i_*), with L = Σ(*l*_i_), and:


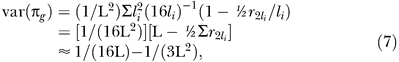


because there are 22 autosomes and r_2*li*_ ≈ ½. These results are the same as those of Guo [11], whose derivations were based upon Markov chains. They imply, that to a first order approximation, the variance in genome-wide IBD sharing is a function of the total genome length only [12,36,38,39]. For L = 35 Morgan, the SD of genome-wide IBD sharing is approximately 0.039. Table 3 shows a breakdown in the variance of IBD sharing per chromosome and the equivalent number of independent loci. It was constructed using the above equations, with physical and genetic lengths from [41], and using the sex-averaged recombination map. For comparison, the SD of the proportion of alleles shared at a given locus is 0.354.

### Dominance.

Dominance variance is a function of the probability that two siblings share both alleles IBD (= IBD2). In a non-inbred population, this probability is also called the coefficient of fraternity [2]. The prior probability that full sibs share two alleles IBD is ¼, and the mean and variance of an indicator variable that is one if both alleles are shared IBD and zero otherwise is ¼ and ^3^/_16_, respectively. Note that the variance of IBD2 sharing at a single locus is 1.5 times the variance of mean IBD sharing. The probability that the sibs share two alleles IBD at a linked locus, given that they are IBD2, is (1 − r)^4^ + 2[(1 − r)r]^2^ + r^4^ = [(1 − r)^2^ + r^2^]^2^. Hence the covariance of the indicator variable (δ) at loci i and j is:


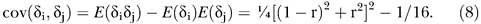


After some algebra it can be shown that the variance of the mean IBD2 sharing (π_di_) on a chromosome of length *l* is:


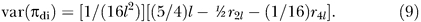


The genome-wide variance in mean IBD2 sharing is:


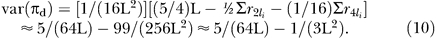


Hence, the variance of the genome-wide IBD2 sharing is larger (by about 30% if L = 35) than the variance of the genome-wide mean IBD sharing. The correlation between mean genome-wide allele sharing and mean genome-wide IBD2 sharing is the ratio of the SD,


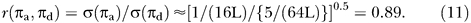


The actual relationship between full sibs can be estimated with genetic markers. For fully informative markers and close relatives, only a few markers are needed per chromosome to capture the proportion of alleles shared IBD [23,24]. This is because the number of recombination events per chromosome is small.

### Sampling variance of estimators of genetic variance.

For *n* sib pairs, the simplest estimation procedure is to apply the Haseman-Elston regression analysis [33] of the squared difference between the phenotypes (Y_i1_ and Y_i2_) of the i^th^ pair of siblings on the estimate of their genome-wide IBD proportion (π_i_),


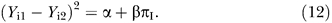


The parameter β is proportional to the within-family additive genetic variance, adjusted for inbreeding in the parents,


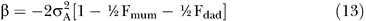


[2,3,33]. We will assume that parents are not inbred, so that the regression slope equals minus twice the additive genetic variance. Then, an estimate of the narrow sense heritability is simply,


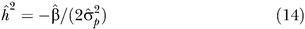


with 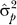 an estimate of the total phenotypic variance. If we ignore the sampling correlation between the estimate of the regression coefficient and the total phenotypic variance, then the sampling variance of the heritability is, using a Taylor series expansion [2]:


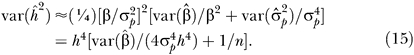


The variance of the regression coefficient is approximately,


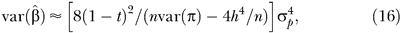


with *t* the sib intra-class correlation [20,21]. Hence, the sampling variance of the estimate of the narrow sense heritability is, approximately,


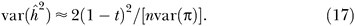


This is fully analogous to the estimation of the proportion of variance explained by a single QTL, the only difference being the variance in genome-wide IBD sharing. The non-centrality-parameter (NCP) for a test of significance of genome-wide additive genetic variance is,


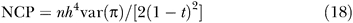


which reduces to the form given by Sham and Purcell [20] and Visscher and Hopper [21] for a single QTL when var(π) = 1/8. Following the derivations in Sham and Purcell [20] and Visscher and Hopper [21], the SE of the estimate of the heritability and NCP when using both the squared difference and squared sum of the sib pairs are, approximately,


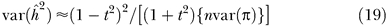


and


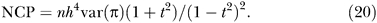


Hence, power calculations for QTL mapping can be used to assess the sample size required to “detect” genome-wide additive genetic variance. For example, to detect a heritability of a given size is equivalent to detecting a QTL at a fully informative locus explaining *q^2^* of the phenotypic variance when *h* ^4^ var(π) = (^1^/_8_)*q* ^4^, i.e., a QTL explaining about 0.11*h^2^* of the phenotypic variance.

ML estimation uses more information than the difference between the sib pairs, and the resulting estimate of the heritability is more accurate. For a single QTL asymptotically (large sample size and a QTL that explains a small amount of variance), the sampling variance of the ML estimator is that of the least squares estimator, when both the squared differences and sums are used in the regression analysis [20,21]. For genome-wide estimation, the proportion of variance explained by π is small (~ 0.11*h* ^2^), so it seems reasonable to use the predictions for the regression analysis. However, the predictions differ dramatically if there is no other source of family resemblance than sharing of genetic effects. The following approximate results were derived assuming the simple equation:


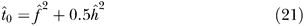


with 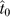 the estimate of the intra-class correlation under the null hypothesis of no genome-wide additive genetic effect, 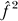 the estimate of the proportion of the variance due to residual familial effects under the alternative hypothesis, and *ĥ* ^2^ the estimate of the heritability under the full model. Equation 21 is a good approximation because the intra-class correlation, which is estimated relatively precisely under the reduced model, is essentially partitioned into a genetic and non-genetic component in the full model. The sampling correlation between the estimates of *f^2^* and *h^2^* is approximately −1. If there are no constraints imposed on the estimates, then, using results from [42],


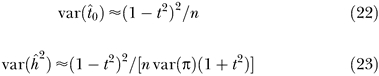


(from [20,21]). By difference,


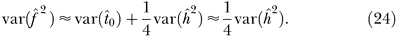


Hence, the SE of the estimate of the non-genetic familial resemblance is approximately half of the SE of the estimate of the genome-wide heritability. The above SE of the estimates can be used to calculate the probability that the ML estimate is zero, using standard normal distribution truncation theory [3] with truncation values of −*f* ^2^/σ(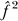) and −*h* ^2^/σ(*ĥ* ^2^), respectively. This was validated using simulations (unpublished data).

### Conditional on *f^2^* = 0.

When the true residual familial component is zero, the ML estimate is zero with a probability of ½, and > 0 with a probability of ½ [27,28]. When the estimate of *f^2^* = 0 then the estimate of the heritability is approximately twice the intra-class correlation of the sibs. Hence, asymptotically,


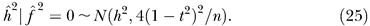


When the estimate of *f^2^* > 0, the mean estimate of the familial component is, approximately,


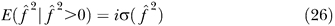


with *i* the mean value of a truncated standard normal distribution. For a truncation value of 0, as is the case here, *i* = 0.798 [3]. The variance of the truncated distribution is:


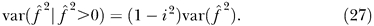


Taking the whole of the distribution of the estimate of *f^2^* gives the mean and variances as:


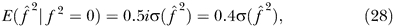


and


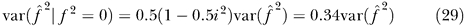


Similarly for the estimate of the heritability,


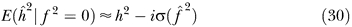


and


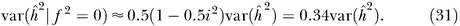


Equations 23, 30, and 31 were used to predict the mean and SE of the estimate of the heritability and were found to be close to simulation results for large samples (Table 1). For small samples the distribution of the estimates of the two variance ratios could be approximated by a truncated bivariate distribution. This situation is more complex because the probability that either estimate is zero as well as the probability that the estimates are constrained at unity needs to be considered jointly. If there is no residual non-genetic family resemblance then the SE of the estimate of the heritability is nearly halved relative to the case where such effects are present. The case of no residual family resemblance is very unlikely for QTL mapping (where the effects of genes elsewhere in the genome and common environmental effects cause resemblance) but realistic for genome-wide analysis of highly heritable phenotypes. The reduction in SE is at the expense of a downward bias in the estimate of the heritability.

### Models.

The basic additive genetic model, fitted in both the simulation study and data application, is Y_ij_ = μ + F_i_ + A_ij_ + E_ij_, with μ the fixed effects of the mean and F, A, and E the random effects of non-genetic family, additive genetic, and residual factors, respectively. The covariance between the phenotypes of two siblings is modeled as cov(Y_i1_,Y_i2_) = var(F_i_) + cov(A_i1_,A_i2_) = σ_F_^2^ + π_a(i)_σ_A_^2^, and cov(Y_ij_,Y_kl_) = 0 if *i* ≠ *j*. Extensions to non-additive models are straightforward, in principle. For example, the covariance for a model containing dominance (D) and additive-by-additive (AA) effects is: cov(Y_i1_,Y_i2_) = σ_F_^2^ + π_a(i)_σ_A_^2^ + π_d(i)_σ_D_^2^ + π_a(i)_π_a(i)_σ_AA_^2^.

### Simulation.

Simulations were performed to validate the predictions of the sampling variance of the heritability and statistical power. Genome-wide IBD sharing between pairs of sibs and their phenotypes were simulated from a simple model,


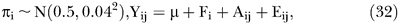


with μ, F, A, and E defined as before, with distributions


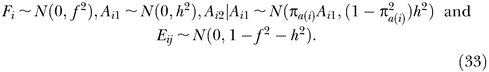


Regression and ML analyses were performed (for details, see [21]). The number of pairs (*n*) in the simulation was either 2,500 or 10,000; heritability values were 0.4, 0.6, and 0.8; and the proportion of variance due to non-genetic family effects was either 0.0 or 0.2. For each set of population parameters, 1,000 replicates were run. Power was calculated using Web-based software for power of QTL analysis [43] at a type-I error rate of 0.05, which is appropriate because we performed a single hypothesis test.

### Application to data.

We estimated the mean and variance of genome-wide IBD sharing from 4,401 quasi-independent full-sib pairs, and applied the ML estimation method to 3,375 quasi-independent full-sib pairs with both marker data and phenotypic measurements on height. These data were collected from two cohorts of Australian twins and their siblings. Phenotypes for the adolescent cohort were collected in the context of continuing longitudinal studies examining risk factors for melanoma [44] and cognitive functioning [45]. For this cohort, height was measured during a clinical examination using a stadiometer at ages 12, 14, and 16; the most recent measurement being used in the current analyses. In the first instance phenotypes for the adult cohort (consisting of twins registered with the Australian Twin Registry born prior to 1971) were collected from self-report questionnaires. Through their subsequent participation in a variety of studies, 58% of the twins included here attended a clinical examination in which height was measured using a stadiometer [15,46]; self-reported height was analyzed if no clinical measurement existed. Correlation between clinically measured and self-reported height was 0.92 in individuals measured both ways [15]. Age at time of measurement was used as a covariate in both cohorts.

Genotypic information was available for a subset of the adolescent and adult participants. For the adolescent cohort, genotypic information was available for 1,201 individuals from 500 families, yielding 950 quasi-independent full-sib pairs. Genotypic information was available for up to 791 autosomal markers. The number of markers per participant in the current study ranged from 211 to 791, with a mean and SD of 588 and 194, respectively, giving an average marker spacing of 6 cM per genotyped individual. The genotyping, error checking, and cleaning of these data have been described in detail elsewhere [47]. For the adult cohort, genotypic information was available for 3,804 individuals from 1,512 families, yielding 3,451 quasi-independent full-sib pairs. Genotypic information was available for up to 1,717 autosomal markers. The number of markers per participant in the current study ranging from 201 to 1,717, with mean and SD of 628 and 264, respectively, and the average marker spacing was 5.6 cM per individual. Details of the genotyping, error checking, and cleaning strategies of these data are given elsewhere [48].

Phenotypes for height were missing on 481 individuals, eight in the adolescent cohort and 473 in the adult cohort. The number of sib pairs for which both individuals had a measured phenotype for the adolescent cohort, the adult cohort, and the combined cohort was 931, 2,444, and 3,375, respectively.

IBD probabilities at 1 cM intervals were calculated using Merlin [49], and the estimate of chromosome and genome-wide IBD sharing was enumerated by averaging the IBD probabilities over the length of a chromosome and the whole genome, respectively.

Each dataset was first adjusted for fixed effects, using a general linear model in which sex was fitted as a fixed factor and age at measurement as a linear covariate. Residuals from this analysis were standardized by the residual variance for each dataset because there was some evidence of heterogeneity of variance: the residual SD for the adolescent and adult dataset was 7.71 cm and 6.89 cm, respectively.

ML analysis was performed using Mx [4]. The full model, termed FAE, contained F and A and E. The covariance between the phenotypes of sibs one and two of pair *i* was modeled as cov(Y_i1_,Y_i2_) = σ_F_^2^ + π_a(i)_σ_A_^2^, with π_a(i)_ the estimate of the genome-wide actual additive relationship of the sibling pair. Reduced models FE and AE were subsequently fitted. A likelihood-ratio-test was performed to test the null hypothesis that A was zero, by comparing the MLs of models FAE and FE. A *p*-value was calculated assuming that the test statistic has an asymptotic distribution that is 0 with a probability of ½ and a one degree of freedom χ^2^ with a probability of ½ [27,28]. CIs of the variance ratios were calculated by Mx and verified by a profile likelihood approach, in which one variance component at a time was changed from its ML value, while maximizing the likelihood for the remaining parameters, until a drop in twice the log-likelihood of 3.84 was reached. In addition to estimating the ML estimate of the variance components for F, A, and E, the ML estimate of (F + A) and its 95% CI were estimated. This was performed because the estimates of F and A have a large negative sampling correlation, so that the estimate of their sum is more precise than the estimate of the individual components.

## Abbreviations

A: genome-wide additive effect
CI: confidence interval
DZ: dizygotic
E: residual error effect
F: non-genetic family effect
FAE: full model
FE: reduced model, containing F and E effects only
IBD: identical-by-descent
IBD2: identical-by-descent at two alleles
ML: maximum likelihood
MZ: monozygotic
SD: standard deviation
SE: standard error
QTL: quantitative trait loci
